# Carum induced hypothyroidism: an interesting observation and an experiment

**DOI:** 10.1186/s40199-015-0094-9

**Published:** 2015-01-24

**Authors:** Seyede Maryam Naghibi, Mohamad Ramezani, Narjess Ayati, Seyed Rasoul Zakavi

**Affiliations:** Nuclear Medicine Research Center, Faculty of Medicine, Mashhad University of Medical Sciences, Mashhad, Iran; Pharmaceutical Research Center, Buali Research Institute, Mashhad University of Medical Sciences, Mashhad, Iran

**Keywords:** Thyroid, Carum carvi, TSH, Hypothyroidism

## Abstract

*Carum carvi* is a widely available herb that has been used as a food additive and as a medication in traditional medicine for many years. Its potential biological effects include analgesic, anti-inflammatory, anti-anxiety and antispasmodic activities. We report a patient with papillary thyroid carcinoma who were under treatment with levothyroxine and experienced an elevated TSH level by ingestion of *Carum carvi*. TSH level was increased to 60.3 mIU/L with no change in levothyroxine dosage and decreased to normal range after discontinuation of the *Carum carvi*. Observing this dramatic change in TSH level by carum ingestion, carum carvi capsules was produced and one of the researcher tried the medication on herself with a dose of 40 mg/kg/day. She had a history of hypothyroidism and was taking 100 ugr/day of levothyroxine. TSH was markedly increased 2 weeks after ingestion of Carum carvi and returned to normal range 5 months after discontinuation of it. This case report shows the effect of consumption of Carum carvi in increasing TSH level in hypothyroid patients treating with levothyroxine. The exact mechanism of action of carum carvi remains unknown.

## Background

Medicinal plants play a key role in several physiologic and pathologic processes in our body and there are many effects which are still to be discovered. *Carum carvi* (black zeera) from Apiaceae family has various biological effects including analgesic, anti-inflammatory, anti-anxiety and antispasmodic activities which provide pharmacological basis for its use in hyperactivity disorders of gut and airways, such as diarrhea, colic and asthma [1-9]. It seems to be a safe food additive commonly used in our daily life but its effect on thyroid hormones remains unknown.

In this study we are reporting two patients with hypothyroidism, under treatment with levothyroxine, who showed elevated TSH levels after ingestion of Carum carvi. This is the first report on effect of carum on thyroid hormones level in patients treating with levothyroxine.

## Case presentation

The first case was a 24-years-old girl (weight: 44.2 kg) with advanced papillary thyroid cancer (T4N1bM1) who had a history of near total thyroidectomy, external radiotherapy of the neck and mediastinum and repeated radio-iodine therapy in the last 17 years. She had bilateral lung metastases and received 34.4 GBq of I-131 during her treatment. She was under suppressive therapy with 100 μg/day of levothyroxine. She was also taking calcium carbonate 1000 mg/day and calcitriol 0.25 μg/day. Her TSH level ranged from 0.07-0.3 mIU/L during follow up consistent with subclinical hyperthyroidism. During her last follow up, TSH and T3RIA level were found to be 60.3 μU/L and 135.9 ng/ml, respectively. Padyab Teb kits was used for measurement of TSH (interassay CV = 8.21%, Intra-assay CV = 5.41%, Padyab Teb, IRI) and T3RIA (interassay CV = 5.56%, intra-assay CV = 5.45%). The laboratory results were repeated and TSH and T3RIA level were 60.72 mIU/L and 150.2 ng/ml, respectively. She had no complaint except occasional cough and dyspnea which were due to bilateral lung metastasis. Biochemically, she was considered hypothyroid. Complete interview was done to find the reason behind the elevated TSH level during levothyroxine consumption. The patient was not receiving any new medication and her drug regimen had not been changed during last 6 months. The only new food additive, she was receiving was *Carum carvi* seed and yarrow. Dose of levothyroxine was not changed and *Carum carvi* and yarrow consumption was stopped. After 2 months of discontinuation of *Carum carvi* and yarrow consumption, measurement of thyroid hormones was performed using the same kits in the same laboratory. The new TSH and T3RIA levels were 1.75 mIU/l and 166.07 ng/ml, respectively indicating euthyroid state. This observation was assessed by Naranjo causality index and found a “possible adverse drug reaction” (score 3) while using WHO-UMC causality scale it was considered a “probable” ADR. Written informed consent was obtained from the patient for publication of this Case report and any accompanying images.

Considering the dramatic change in TSH level by carum ingestion, *Carum carvi* capsules containing 200, 400 and 800 mg of powdered *Carum carvi* was produced in School of Pharmacy and one of the researchers with history of hypothyroidism tried to test it on her. She was a 24-years-old girl (weight = 45 kg) with a history of hypothyroidism for 5 years and had been treating with 100 μg/day of levothyroxine. TSH level was between 2.5-3.7 mIU/l in her medical records. In the first visit, all clinical signs and symptoms were assessed and none of hypothyroid symptoms were observed. Her pulse rate was 76/min and her blood pressure was 90/70 mmHg. In physical examination, she had a painless, small goiter with no palpable nodule or adenopathy. She was not taking any other medication and her mother had a history of hypothyroidism as well. Her initial TSH level before starting Carum carvi was 2.3 mIU/L, indicating euthyroidism. She started to take carum with dose of 1800 mg/day (40 mg/kg) divided in 3 doses. The dose of 40 mg/kg was 1% of the maximum safe dose of *Carum carvi* in rats. Levothyroxine was ingested in fasting state in early morning and carum capsules were used after each meal (breakfast, lunch and dinner). After 2 weeks, TSH level was 26 mIU/l and thyroid hormone levels were decreased indicating hypothyroid state (Table 1). Vital signs remained in normal range (PR: 80 and BP: 90/70). In physical examination, no new change was observed but she suffered from dry and cold skin. She continued using carum capsules for 4 additional weeks when she started to complain from constipation. At the same time, TSH level increased to 110 mIU/L, and thyroid hormone level was further decreased (Table 1). Thyroid exam was unchanged while she was in hypothyroid state both biochemically and clinically. *Carum carvi* was discontinued and thyroid values was measured after 2 weeks and showed TSH level of 25 mIU/L. Dose of Levothyroxine was not changed and thyroid values were measured again 6 weeks after discontinuation of *Carum carvi*. The TSH level further decreased to 11 mIU/l and T4RIA level was increased to 9 ng/L (Table 1). Two months later, TSH was further decreased to 7 mIU/l with no intervention indicating subclinical hypothyroid state. The patient obtained 7 scores in Naranjo causality algorithm (probable ADR) and categorized as “certain ADR” in WHO-UMC score.

**Table 1:** **Hormonal levels in different time points after carum ingestion and discontinuation**

	**Carum ingestion**			**Carum discontinued**	
**Time**	**Beginning**	**2 weeks**	**6 weeks**	**8 weeks**	**12 weeks**
TSH (mIU/L)	2.3	26	110	25	11
T4RIA (ng/L)	11	12	3.7	4.6	9
T3RIA (ng/L)	148	106	57	90	143
T3RU (%)	30	31	26	24	30

TSH = Thyroid stimulating hormone, T4RIA = T4 Radio-immuno-assay, T3RIA = T3-Radio-immuno-assay, T3RU = T3 Resin uptake.

## Discussion

In the present study, we observed dramatic effect of oral administration of *Carum carvi* seed on TSH level in patients with hypothyroidism. Although one of our patients had follicular thyroid cancer and the other had hypothyroidism due to autoimmune thyroiditis, both patients was receiving levothyroxine and showed marked elevation of TSH level after ingestion of carum seeds.

*Carum carvi* has been used traditionally as an acceptable food additive and its beneficial effect was reported in treating diarrhea, colic and asthma. Due to its antispasmodic activity, it was used in gastrointestinal disorders and as bronchodilator in hyperactive airways [1,6]. In addition, studies indicated that caraway oil probably has a protective antioxidant role in heart and kidney in patients with sepsis [10]. The mechanism of the effect of *Carum carvi* in our patients is unknown. Our first patient was receiving carum and yarrow while the second patient was receiving carum only. As the effect was similar in both patients, it seems that carum effect is more prominent than any possible effect of yarrow.

Some herbal products were reported to change thyroid metabolism. Soy consumption is related to thyroid disorders such as hypothyroidism, goiter, and autoimmune thyroid disease besides increased iodine metabolism in animal studies but the exact molecular mechanisms which might be responsible for hypothyroidism is unclear [11]. In our patients, hypothyroidism could not be related to iodine metabolism as both patients were taking levothyroxine. It was found that the peel extracts of *Mangifera indica*, *Citrullus vulgaris*, and *Cucumis melo* were thyro-stimulatory increasing the levels of both T3 and T4 which is indicative of the role of these peel extracts in ameliorating hypothyroidism [12,13]. The possible reasons for this beneficial role may be attributed to the presence of flavonoids, phenolic compounds, and ascorbic acid content of the peel extracts [12,14]. Full phytochemical analysis of both essential oil and seed extract showed the presence of many mono and sesquiterpenes in essential oil and diverse flavonoids, isoflavonoids, flavonoid glycosides, monoterpenoid glucosides, lignins and alkaloids and other phenolic compounds in seed extract [13,15-17]. However, none of these compounds has been specifically linked to a mechanism by which TSH elevation could be explained.

In our patient, the mechanism of TSH elevation may be attributed to prevention of absorption of levothyroxine in the bowel and/or its action in the receptor level. Since in our trial case, T4 and T3 levels decreased by consumption of *Carum carvi*, the most plausible explanation would be the interference with levothyroxine absorption. This hypothesis is also supported by the observation that hypothyroidism was gradually becoming severe and symptoms of hypothyroidism were more prominent at the end of the experiment. Interestingly, symptoms and signs of hypothyroidism were not prominent in our patient early after experiment, suggesting that peripheral action of the hormone is well preserved. Moreover, this finding may suggest that Carum may have more prominent effects on blocking of T4 to T3 conversion at the hypophysis.

Many causality scoring systems have been used for evaluation of adverse drug reaction. We used Naranjo score and WHO-UMC in our patients and it was “probable” and “certain” ADR according to these categories, respectively [18].

The carum seeds consumption did not produce any observable toxicity based on evaluated signs and symptoms at the dose of 40 mg/kg [3]. However, more extensive toxicity assessments are required to make sure that the seeds are safe at the dose of 40 mg/kg.

## Conclusion

This case report shows that consumption of *Carum carvi* interfere with levothyroxine effect in hypothyroid patients and increase TSH prominently. The exact mechanism of action of *Carum carvi* is not known. Informing patients and physicians about the possible interaction of *Carum carvi* on TSH elevation would be helpful.

## Abbreviations

TSH: Thyroid stimulating hormone
T3RIA: T3 radioimmunoassay
IRMA: Immuno-radio-metric-assay
GBq: Giga becquerel
CV: Coefficient of variation
ADR: Adverse drug reaction
WHO-UMC: World Health Organization- Upssala Monitoring Center

## References

[1] Khan M, Khan AU, Najeeb u R, Gilani AH (2012). Gut and airways relaxant effects of Carum roxburghianum. J Ethnopharmacol.

[2] Saghir MR, Sadiq S, Nayak S, Tahir MU (2012). Hypolipidemic effect of aqueous extract of Carum carvi (black Zeera) seeds in diet induced hyperlipidemic rats. Pak J Pharm Sci.

[3] Rezvani ME, Roohbakhsh A, Mosaddegh MH, Esmailidehaj M, Khaloobagheri F, Esmaeili H (2011). Anticonvulsant and depressant effects of aqueous extracts of Carum copticum seeds in male rats. Epilepsy Behav.

[4] Sadiq S, Nagi AH, Shahzad M, Zia A (2010). The reno-protective effect of aqueous extract of Carum carvi (black zeera) seeds in streptozotocin induced diabetic nephropathy in rodents. Saudi J Kidney Dis Transpl.

[5] Hejazian YS, Dashti RM, Mahdavi SM, Qureshi MA (2009). The effect of Carum Copticum extract on acetylcholine induced contraction in isolated rat’s ileum. J Acupunct Meridian Stud.

[6] Boskabady MH, Alizadeh M, Jahanbin B (2007). Bronchodilatory effect of Carum copticum in airways of asthmatic patients. Therapie.

[7] Dashti-Rahmatabadi MH, Hejazian SH, Morshedi A, Rafati A (2007). The analgesic effect of Carum copticum extract and morphine on phasic pain in mice. J Ethnopharmacol.

[8] Lemhadri A, Hajji L, Michel JB, Eddouks M (2006). Cholesterol and triglycerides lowering activities of caraway fruits in normal and streptozotocin diabetic rats. J Ethnopharmacol.

[9] Gilani AH, Jabeen Q, Ghayur MN, Janbaz KH, Akhtar MS (2005). Studies on the antihypertensive, antispasmodic, bronchodilator and hepatoprotective activities of the Carum copticum seed extract. J Ethnopharmacol.

[10] Dadkhah A, Fatemi F (2011). Heart and kidney oxidative stress status in septic rats treated with caraway extracts. Pharm Biol.

[11] Tran L, Hammuda M, Wood C, Xiao CW (2013). Soy extracts suppressed iodine uptake and stimulated the production of autoimmunogen in rat thyrocytes. Exp Biol Med (Maywood).

[12] Parmar HS, Kar A (2008). Possible amelioration of atherogenic diet induced dyslipidemia, hypothyroidism and hyperglycemia by the peel extracts of Mangifera indica, Cucumis melo and Citrullus vulgaris fruits in rats. Biofactors.

[13] Parmar HS, Kar A (2009). Protective role of Mangifera indica, Cucumis melo and Citrullus vulgaris peel extracts in chemically induced hypothyroidism. Chem Biol Interact.

[14] Parmar HS, Kar A (2008). Antiperoxidative, antithyroidal, antihyperglycemic and cardioprotective role of Citrus sinensis peel extract in male mice. Phytother Res.

[15] Ishikawa T, Takayanagi T, Kitajima J (2002). Water-soluble constituents of cumin: monoterpenoid glucosides. Chem Pharm Bull (Tokyo).

[16] Matsumura T, Ishikawa T, Kitajima J (2002). Water-soluble constituents of caraway: aromatic compound, aromatic compound glucoside and glucides. Phytochemistry.

[17] Takayanagi T, Ishikawa T, Kitajima J (2003). Sesquiterpene lactone glucosides and alkyl glycosides from the fruit of cumin. Phytochemistry.

[18] Belhekar MN, Taur SR, Munshi RP (2014). A study of agreement between the Naranjo algorithm and WHO-UMC criteria for causality assessment of adverse drug reactions. Indian J Pharmacol.

